# *LIM Homeobox 9* knockdown by morpholino does not affect zebrafish retinal development

**DOI:** 10.1242/bio.056382

**Published:** 2021-03-08

**Authors:** Rui Guo, Fei Li, Minxia Lu, Kangkang Ge, Lin Gan, Donglai Sheng

**Affiliations:** 1 Key Laboratory of Organ Development and Regeneration of Zhejiang Province, College of Life and Environmental Sciences, Hangzhou Normal University, Hangzhou 311100 Zhejiang, China; 2 College of Life Sciences, Zhejiang University, Hangzhou 310013 Zhejiang, China; 3 Hangzhou jingbai biotechnology Co, LTD., Hangzhou 310004 Zhejiang, China

**Keywords:** Lhx9, Expression, Zebrafish, Retina, Morpholino

## Abstract

LIM homeobox 9 (Lhx9) is a member of the LIM homeodomain transcription factor family, which expresses and functions in various vertebrate tissues, such as the gonads and pineal gland. Previous studies on *lhx9* in zebrafish have mainly focused on the brain. However, little is known about the expression pattern of *lhx9* during embryogenesis. Here, we detected *lhx9* expression in zebrafish embryos using whole-mount *in situ* hybridization and found *lhx9* expressed in heart, pectoral fin, and retina during their development in zebrafish. We then detailed the expression of *lhx9* in retinal development. To further investigate the function of Lhx9 in retinogenesis, we performed morpholino (MO) knockdown experiments and found that upon *lhx9* knockdown by MO, larvae presented normal eye development, retinal neural development, differentiation, proliferation, apoptosis, and responses to light stimulus. We not only elaborated the expression pattern of *lhx9* in zebrafish embryogenesis, but we also demonstrated that *lhx9* knockdown by morpholino does not affect the zebrafish retinal development, and our study provides data for further understanding of the role of Lhx9 in zebrafish retinal development.

## Abstract (precis)

**Summary:** *lhx9* is expressed in the development of the zebrafish heart, pectoral fin, and retina, but *lhx9* knockdown by morpholino does not affect zebrafish retinal development.

## INTRODUCTION

The vertebrate retina, a part of the central nervous system (CNS), is a highly conserved structure. The zebrafish retina is an ideal model to study the development of the CNS (DeCarvalho et al., 2004; Malicki et al., 1996; Meier et al., 2018; Vitorino et al., 2009). The mature zebrafish retina consists of six types of neurons and Müller glial cells (MGCs), which formed three cellular layers and two plexiform layers. The outer nuclear layer (ONL) contains photoreceptors (PRs), including rod and cone photoreceptors (Lu et al., 2019; Shi et al., 2017). Meanwhile, the inner nuclear layer (INL) contains horizontal cells (HCs), bipolar cells (BCs), amacrine cells (ACs), and MGCs (Haug et al., 2019). Retinal ganglion cells (RGCs) are present in the ganglion cell layer (GCL) (Mu et al., 2017). Synapses connecting the terminals of PRs and dendrites of BCs are found in the outer plexiform layer (OPL) (McGinn et al., 2018), and synapses contacting BCs or ACs with RGCs are present in the inner plexiform layer (IPL) (Chow et al., 2015).

In the different stages of vertebrate retinogenesis, many transcription factors (TFs) are intrinsically involved. The expression and function of some LIM-homeodomain (LIM-HD) TFs in the retina have been reported (Blixt et al., 2018; Zagozewski et al., 2014). In the developing mouse retina, Lhx1 (as referred to as Lim1) is expressed in postmitotic HCs and mainly takes part in HC location (Edqvist and Hallböök, 2004; Liu et al., 2000). Overexpression of *Lhx1* in the chick optic vesicle enhances the expression of retinal specific genes (Kawaue et al., 2012). In the developing mouse retina, *Lhx2* is expressed in the prospective eye field. Knockout of the mouse *Lhx2* results in anophthalmia (Tétreault et al., 2009; Yun et al., 2009). However, probably due to the functional redundancy with *lhx2* ortholog *lhx2a* in zebrafish, mutation of *lhx2b* does not induce anophthalmia (Seth et al., 2006). In the developing *Xenopus* retina, *Lhx9* is expressed in the INL neurons (Atkinson-Leadbeater et al., 2009). In the developing mouse retina, the expression of *Lhx9* starts to appear in the retinal neuroblast layer (NBL) at E13.5, and it continues to be expressed in the INL and GCL of the adult retina (Balasubramanian et al., 2014, 2018). *Lhx9* knockout in the mouse retina reduces neural retinal specific gene expression (Balasubramanian et al., 2018). However, the expression pattern and function of Lhx9 are not well understood in the zebrafish retina.

In this study, we aimed to reveal the detailed expression pattern of *lhx9* in zebrafish embryogenesis and retinogenesis by whole-mount *in situ* hybridization (WISH) and section *in situ* hybridization (ISH). Furthermore, we performed a morpholino (MO) *lhx9* knockdown experiment in zebrafish and examined the effects of *lhx9* knockdown on retinal neuronal differentiation, proliferation and apoptosis, and the responses to light stimulus.

## RESULTS

### Expression pattern of *lhx9* in zebrafish embryogenesis and retinogenesis

**Fig. 1.**
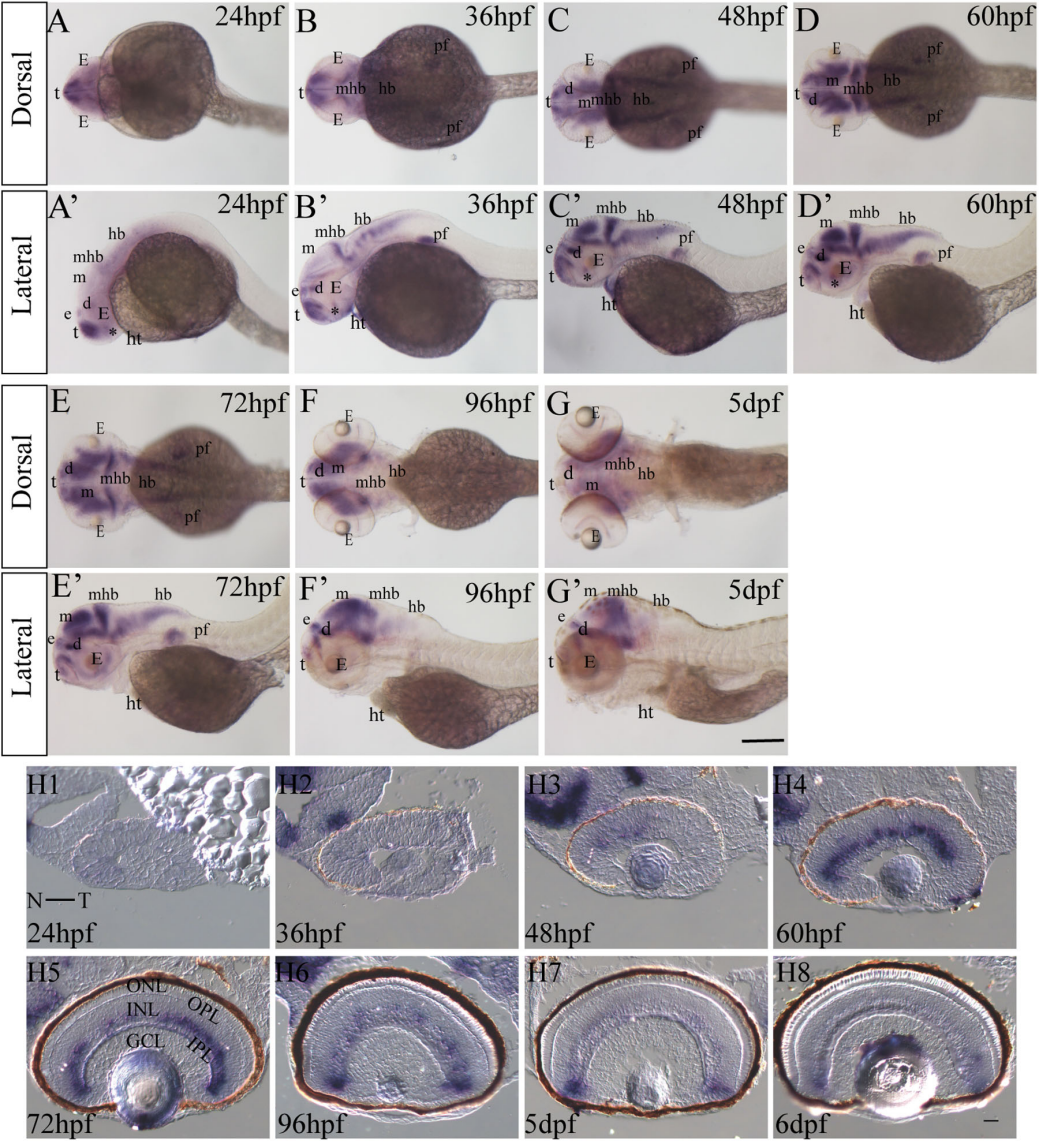
**Expression pattern of *lhx9* in zebrafish embryogenesis and retinogenesis.** (A–G′) *lhx9* expressed in the zebrafish embryo at 24 hpf, 48 hpf, 60 hpf, 72 hpf, 96 hpf, and 5 dpf. t, telencephalon; E, eye; e, epiphysis; d, diencephalon; m, mesencephalon; mhb, mid-hindbrain boundary; hb, hindbrain; ht, heart; pf, pectoral fin; asterisk, hypothalamus; Scale bar, 500 μm. (H1–H8) The expression of *lhx9* in the zebrafish retina at 24 hpf, 36 hpf, 48 hpf, 60 hpf, 72 hpf, 96 hpf, 5 dpf, and 6 dpf. Figures are horizontal sections along the temporal-nasal axis (T–N). ONL, outer nuclear layer; INL, inner nuclear layer; GCL, ganglion cell layer; OPL, outer plexiform layer; IPL, inner plexiform layer. Scale bar=20 μm.

Previous studies have reported that, spatiotemporally, the expression of *lhx9* is fragmentary in zebrafish embryogenesis, observed predominantly in the brain (Ando et al., 2005; Liu et al., 2015; Peukert et al., 2011). To clearly answer this question, we detected *lhx9* expression using WISH in zebrafish embryos from 24 hours past fertilization (hpf) to 5 days past fertilization (dpf) and it was detected in the dorsal and lateral views. *lhx9* expression is detected in the brain at 24 hpf, including in the telencephalon, diencephalon, epiphysis, hypothalamus, mesencephalon, mid-hindbrain boundary, and hindbrain (Fig. 1A,A′). The expression of *lhx9* in these regions increases and is maintained at a relatively high level in the following stages (Fig. 1B–G′). In addition, we detected that *lhx9* expression begins in the heart and pectoral fin at 36 hpf (Fig. 1B′), and continues up to 60 hpf and 72 hpf, respectively (Fig. 1C–E′), while the expression in the retina begins at 48 hpf (Fig. 1D,D′) and continues up to the last stage (5 dpf) by us (Fig. 1E–G′). The expression of *lhx9* was almost undetectable in the embryos using the sense probe (data not shown). These results indicate that *lhx9* not only plays an important role in brain development, but is also likely to play a role in heart, pectoral fin, and retina development.

To further investigate the localization of the *lhx9* transcript in the retina, we used section ISH. These results were consistent with WISH, where *lhx9* was weakly expressed in the ventral region of the NBL at 48 hpf (Fig. 1H3). After the INL and ONL were specified (60 hpf), the *lhx9* transcript expanded to the cells in the INL (Fig. 1H4–H6). The expression of *lhx9* in the retina gradually decreased and focused on the ciliary marginal zone (CMZ) after 5 dpf (Fig. 1H7–H8). The expression of *lhx9* was almost undetectable in retinas using the sense probe (data not shown). These data suggest that Lhx9 might play a role in zebrafish retinogenesis.

### A zebrafish model with *lhx9* knockdown via MO

**Fig. 2.**
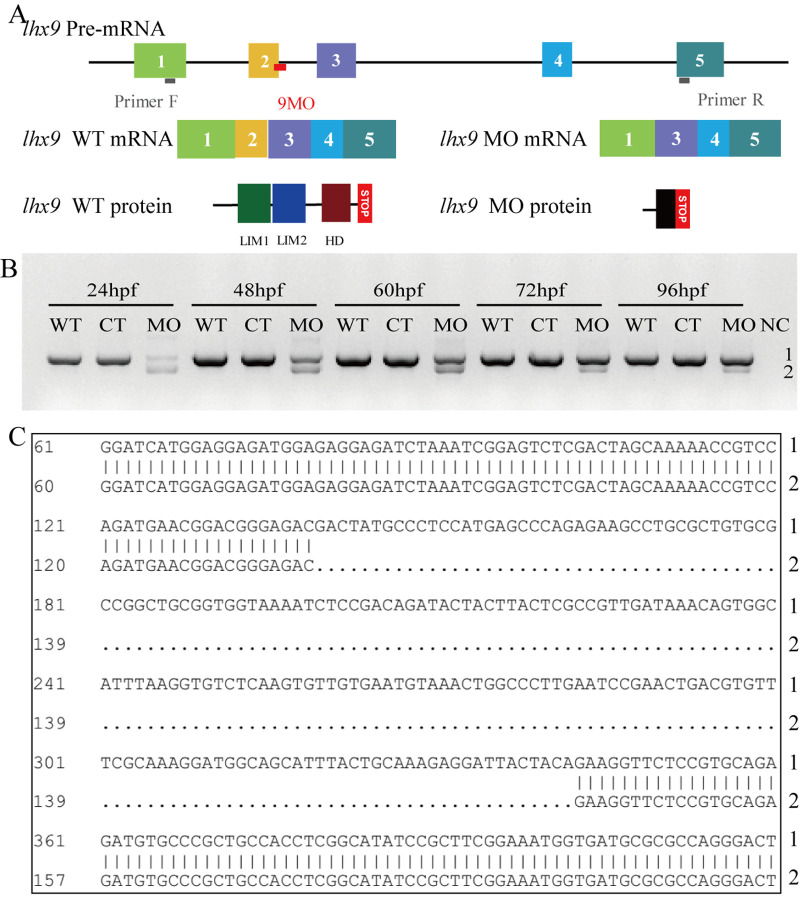
**Identification of the efficiency of *lhx9* MO in the zebrafish embryo.** (A) Schematic diagram of *lhx9* MO blocking *Lhx9* mRNA splicing and protein translation. Exon 2 was deleted in the *lhx9* transcript by MO, the LIM1 domain of Lhx9 protein was removed, and the LIM2 and HD domain can't be assembled because of the wrong amino acid sequences and the early stop codon. (B) RT-PCR confirming the effect of splice-blocking MO on *lhx9* transcript at 24 hpf, 48 hpf, 60 hpf, 72 hpf, and 96 hpf. MO lanes (MO cDNA as the template) show two PCR bands, band 1 and 2 (736 bp). (C) Sequence alignment of band 1 (939 bp) and band 2 (736 bp).

To assess the function of Lhx9 during zebrafish retinal development, a splice-blocking MO was injected into the embryos to knockdown *lhx9* expression. RT-PCR results showed that Lhx9 mRNA in MO embryos produced two PCR bands, band 1 of normal size (939 bp), and band 2 of reduced size (approximately 736 bp; a 203 bp difference), at 24 hpf, 48 hpf, 60 hpf, 72 hpf, and 96 hpf (Fig. 2B). Sequence analysis demonstrated that exon 2 (203 bp) of Lhx9 mRNA was excluded in the mature transcript in MO embryos (Fig. 2C), which can cause a frameshift mutation and eventually result in domain translation failure (Fig. 2A). These data indicated that the MO can effectively knock down the expression of Lhx9 mRNA from 24 hpf to 96 hpf. To validate the efficiency, we tested the expression of *hcrt,* a downstream gene of *lhx9* (Liu et al., 2015), and found it was significantly reduced in MO larvae, compared with wild-type (WT) and control (CT) larvae at 48 hpf (Fig. S1).

Subsequently, the morphology of WT, CT, and MO embryos was assessed at 36 hpf, 48 hpf, and 72 hpf. Compared with WT and CT embryos (Fig. S1A–F), MO embryos showed no apparent malformation (Fig. S2G–I) until 72 hpf. We quantified these data in embryos from three groups. MO (*n*≥15 in each group) embryos showed no significant difference in body length (Fig. S2J–L) and eye size (Fig. S2M–O) compared to WT and CT embryos. Therefore, *lhx9* knockdown by MO does not affect zebrafish body and eye development.

### Effects on the retinal neuronal differentiation

**Fig. 3.**
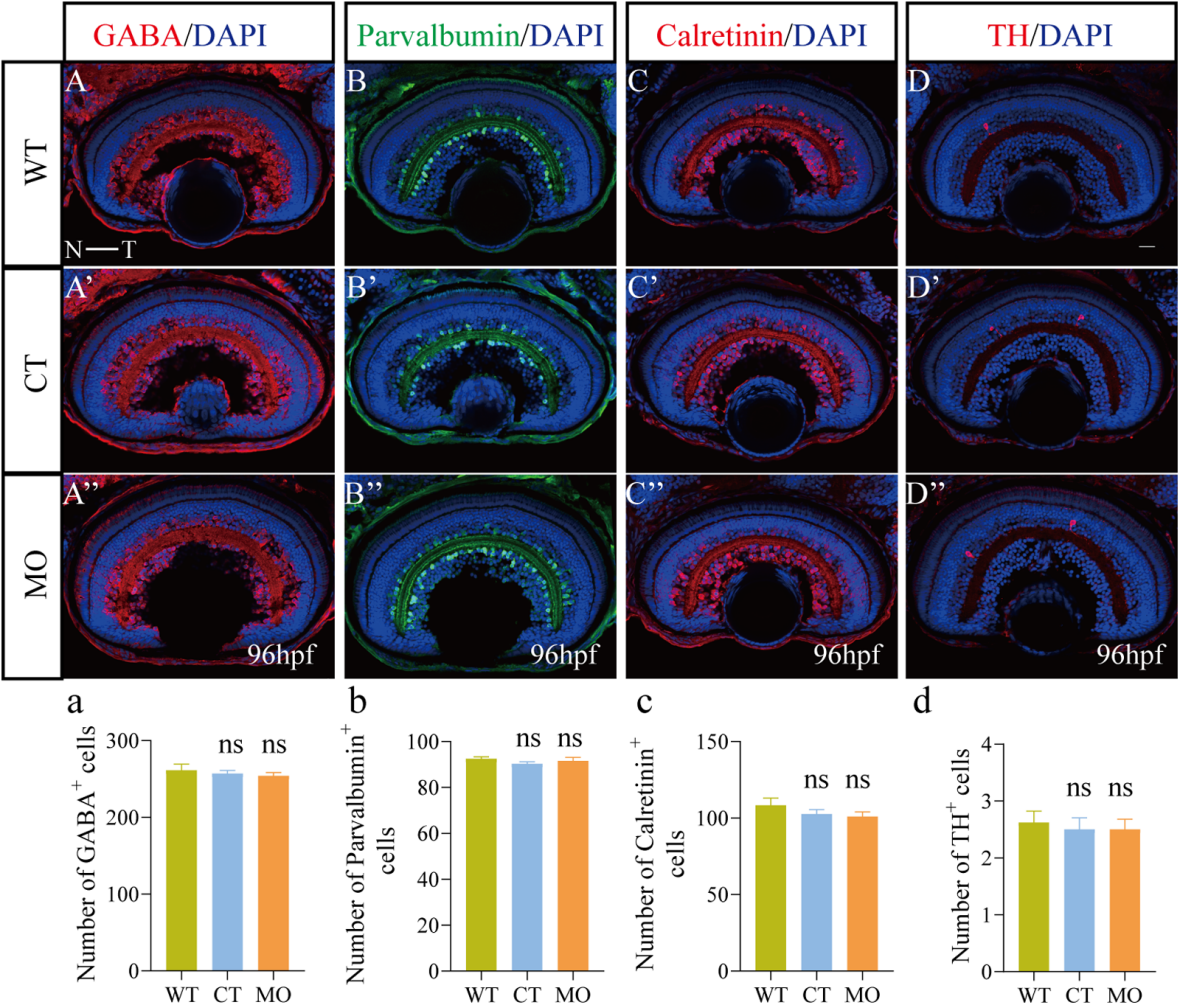
**Immunofluorescence staining analysis of the retinal neuronal differentiation in the INL.** All figures are horizontal sections along the temporal- nasal axis (T-N). (A–D″) Immunofluorescence staining with different ACs markers, GABA, Parvalbumin, Calretinin, and TH, in WT, CT, and MO retinas at 96 hpf. (a-d) statistical analysis of GABA^+^, Parvalbumin^+^, Calretinin^+^ and TH^+^ cells in WT, CT, and MO retinas at 96 hpf. Blue, DAPI staining of the nuclei. Scale bar=20 μm. (a–d) Statistical analysis of GABA^+^, Parvalbumin^+^, Calretinin^+^, and TH^+^ cells in WT (*n*=16), CT (*n*=15), and MO (*n*=16) retinas at 96 hpf. Results are represented as the mean±s.e.m. ns, *P*>0.05; assessed by one-way ANOVA followed by Tukey's multiple comparisons.

We performed immunofluorescence staining and qRT-PCR to explore neuronal differentiation in the retina using various retinal markers. In MO retinas, the expression of AC markers, including GABA (Fig. 3A″), Parvalbumin (Fig. 3B″), Calretinin (Fig. 3C″) and TH (Fig. 3D″), were not different from those in the WT (Fig. 3A–D) or CT group at 96 hpf (Fig. 3A′–D′). Compared with WT and CT retinas, there was no significant difference in the number of these AC subtypes in MO retinas at 96 hpf (Fig. 3A″–D″).

Moreover, we found that the expression of Zn8, a marker of RGCs, was unchanged in MO retinas at 72 hpf (Fig. S3A–A″) or 96 hpf (data not shown). Compared with WT and CT retinas, there was no significant difference in the width of GCL (Zn8^+^ RGCs) in MO retinas at 72 hpf (Fig. S3a). The development of BCs (marked by PKCα) (Fig. S3B–B″), cone PRs (marked by Zpr1) (Fig. S3C–C″), and rod PRs (marked by Rhodopsin) (Fig. S3D–D″) was also normal at 96 hpf. Compared with WT and CT retinas, there was no significant difference in the number of these neurons in MO retinas at 72 hpf (Fig. S3b–d).

In addition, we detected other genes related to retinal development by qRT-PCR, including *prox1*, a marker of HCs; *gs*, a marker of MGCs; *nos*, a marker of ACs; and *vsx2*, a marker of BCs and MGCs. They were unchanged in MO retinas at 96 hpf (Fig. S4). These data indicated that *lhx9* knockdown by MO did not affect neuronal differentiation in the retina.

### Effects on retinal cell apoptosis and proliferation

To test whether *lhx9* knockdown affects cell apoptosis and proliferation in the retinas, we performed immunofluorescence staining with antibodies against active Caspase-3 and PH3. As shown in Fig. S4, the number of apoptotic cells, marked by the antibody against active Caspase-3 (Fig. S5A–B′) and proliferation cells, marked by the antibody against PH3 (Fig. S5C–D′) in the MO retinas were not different from those of CT and WT retinas at 36 hpf or 48 hpf (Fig. S5a-d), indicating that apoptosis and proliferation were normal in the *lhx9* knockdown retina.

### Effects on the responses to light stimulus

**Fig. 4.**
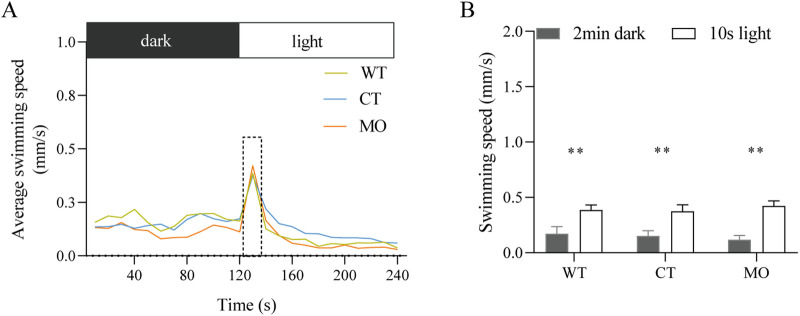
**Response of the zebrafish larvae to the light stimulus at 5 dpf.** (A) The average swimming speed of the larvae during the last 2 min dark and 2 min light period. (B) The swimming speed during the last 2 min dark and the first 10 s of the 2 min light period. Results are represented as the mean±s.e.m. (WT, *n*=35; CT, *n*=34; MO, *n*=32). **P<0.01; assessed by one-way ANOVA followed by Tukey's multiple comparisons.

Based on the above data, we speculated that MO larvae might be as sensitive as CT and WT larvae in their response to a light stimulus. Larvae of the three groups were tested in a behavioral test at 5 dpf. When larvae were immediately exposed to the light after 2 min in the dark (preceded by 30 min darkness), they all exhibited a peak in the first 10 s of the 2 min light period (Fig. 4A). Moreover, all groups showed a significant increase after exposure to the light stimulus (Fig. 4B). These data showed that the responses to the light stimulus were not affected in the larvae injected with *lhx9* MO.

## DISCUSSION

Lhx9 is a member of the LIM-homeodomain (LIM-HD) TFs family, and it is expressed and functions in various vertebrate tissues, such as the gonad and pineal glands (Liu et al., 2015; Peukert et al., 2011; Rétaux et al., 1999) Previous studies on *lhx9* in zebrafish have focused on the brain. The beginning of *lhx9* expression in the zebrafish embryo has been reported in the presumptive forebrain at 11–12 hpf (Ando et al., 2005; Harrison et al., 2011), which is very clear and definite. The report by Liu et al. is the only study that detected the expression of *lhx9* from 24 hpf to 5 dpf, but they only detected it in the hypothalamus (Liu et al., 2015). Hence, the expression pattern of *lhx9*, spatiotemporally, in zebrafish embryogenesis is fragmentary. In this study, we detected *lhx9* expression using WISH in zebrafish embryos from 24 hpf to 5 dpf, and we demonstrated its presence in dorsal and lateral views to clarify this issue. At prim 5 (24 hpf we detected), Ando et al. found that *lhx9* is expressed in the brain, including in the telencephalon, diencephalon, hypothalamus, mid-hindbrain boundary, and hindbrain (Ando et al., 2005). In addition to the above zones, Liu et al. found that *lhx9* was also expressed in the epiphysis at 24 hpf (Liu et al., 2015). At prim 15 (detected as 36 hpf), Nikolaou et al. detected the expression of *lhx9* in the hindbrain (Nikolaou et al., 2009), and Peukert et al. detected it in the forebrain (Peukert et al., 2011). At 48 hpf, Peukert et al. and Yelin-Bekerman, et al. detected the expression of *lhx9* in the brain (Peukert et al., 2011; Yelin-Bekerman et al., 2015). We showed the spatiotemporal expression of *lhx9* in the embryo and detected *lhx9* expression in heart and pectoral fin development in zebrafish, which lasted until 60 hpf, and 72 hpf, respectively.

Moreover, we detailed the expression pattern of *lhx9* in the developing zebrafish retina. The *lhx9* transcript is expressed in the zebrafish retinal neuroblast layer (NBL) beginning at 48 hpf, and has highly expressed in the INL at 60–96 hpf, beyond which, the expression of *lhx9* in the zebrafish retina gradually decreased. The retina is a part of the CNS and is highly conserved among vertebrates. In the developing *Xenopus* retina, *Lhx9* transcript is expressed in the INL neurons (Atkinson-Leadbeater et al., 2009), and *Lhx9* is expressed in the INL and GCL in the mouse retina, (Balasubramanian et al., 2014, 2018). Therefore, the expression pattern of *Lhx9* is largely conserved in the vertebrate retinas.

In zebrafish, eye morphogenesis begins at 12 hpf (Schmitt and Dowling, 1996), and well-formed optic cups, including the inner and outer layer, appear at 24 hpf. As in other vertebrates, in zebrafish, RGCs and ACs first arise at about 32 hpf. PR and BC form at approximately 50 hpf and 60 hpf, respectively (Schmitt and Dowling, 1994). Until 96 hpf, the different cell types in the zebrafish retina can be distinguished. *lhx9* knockdown by MO might influence any of the cell subtypes, although it begins to express at 48 hpf. However, compared with CT and WT retinas, the expression of GCs, BCs, ACs, PRs, MGCs, and HCs in MO retinas showed no significant changes until 96 hpf.

Balasubramanian et al. found that the expression of GABA, NOs, and Calretinin is abnormal in *Lhx9*-null retinas with conditional knockout technology (Balasubramanian et al., 2018). However, we detected no significant difference in MO retina compared with CT or WT retinas. We speculate that the effect of *lhx9* knockdown on the expression of the retinal GABA, NOs, and Calretinin is unequal with *lhx9* knockout. Besides, Peukert et al. reported that *lhx9* and *lhx2* are redundant in the neuronal differentiation of the zebrafish thalamus, and although single knockdown of *lhx9* or *lhx2* has no effect on thalamus neurogenesis, simultaneous knockdown of both *lhx9* and *lhx2* does (Peukert et al., 2011). Thus, the function of *lhx9* may be redundant in the zebrafish retinogenesis.

Recently, the light responses were reported in the analysis of the zebrafish vision function (Cai et al., 2018; Houbrechts et al., 2016; Zhuang et al., 2019). Knockdown of T3-inactivating D3 by MO impaired light responses at 4 dpf and 5 dpf (Houbrechts et al., 2016). *il7^−/−^* larvae exhibited weak light responses at 6 dpf, compared to WT larvae (Cai et al., 2018). It is known that zebrafish larvae have the visual function and swimming ability at 4 dpf and 5 dpf, respectively (Brockerhoff, 2006; Li et al., 2015). Therefore, we analyzed the visual function at 5 dpf in this study. All larvae from WT, CT, and MO groups exhibited a sharp increase in swimming speed when there was a light stimulus. Moreover, the swimming speeds of larvae from the three groups were not different, whether in the last 2 min dark or the first 10 s of 2 min light period. These results indicate that Lhx9 knockdown does not affect the visual function or swimming ability of zebrafish.

MOs have been used widely in zebrafish for many years (Kizil et al., 2013; Pasini et al., 2004; Scholpp et al., 2006). In general, more than 50% of injected embryos exhibit the biologically specific phenotype at doses of 5.0 ng or less, and injection of 6 ng or more sometimes results in embryos displaying off-target effects (Nasevicius and Ekker, 2000; Stainier et al., 2017). In 2011, Peukert et al. efficiently knocked down Lhx9 using MO at a concentration of 0.5 mM, zebrafish displayed a non-splicing of intron 1 and had no diencephalic phenotype (Peukert et al., 2011). Liu et al. demonstrated knockdown of lhx9 reduced the expression of *hcrt* in zebrafish (Liu et al., 2015). In this study, we injected another MO at a dose of 5.0 ng (0.5 mM), causing a reading frameshift mutation (exon 2 deleted) from 24 hpf to 96 hpf and decreased the expression of *hcrt*. However, we found that *lhx9* knockdown had no effect on retinal development. In the future, we will verify the effect of *lhx9* knockout in zebrafish using CRISPR/Cas9 technology.

In summary, we detailed the spatiotemporal expression pattern of *lhx9* in zebrafish embryo development. Furthermore, we found that zebrafish Lhx9 knockdown by MO does not affect eye growth, retinal neuron development, differentiation, proliferation, apoptosis, or the responses to light stimulus. This study provides data for further understanding of the role of Lhx9 in zebrafish retina development.

## MATERIALS AND METHODS

### Animals

The WT/AB zebrafish (*Danio rerio*) used in the study were treated according to the guidelines for animal use and care in Hangzhou Normal University. Zebrafish were raised at 28°C under a 10 h:14 h dark/light cycle. Zebrafish embryos were raised in E3 medium at 28°C and developmentally staged by hpf or dpf.

### MO knockdown experiment

Two MOs used in the study were obtained from Gene Tools LLC (Philomath, OR, USA): a splice-blocking *lhx9* MO (GCC TCA AAG TTA ATG CTT ACC TGT A), and a control 5-bp mismatch MO (GCg TgA AAc TTA ATc CTT ACC TcT A). Every embryo in the MO or CT group was injected with 5.0 ng *lhx9* MO or 5-misMO. All CT and MO embryos were injected side by side at the one- to four-cell stages. The WT group embryos were raised under the same conditions without any treatment.

To confirm the efficiency of *lhx9* MO, total RNA was extracted from the whole embryo of three groups at 24 hpf, 48 hpf, 60 hpf, 72 hpf, and 96 hpf, using TRIzol reagent (Sangon Biotech, Shanghai, China). cDNA was obtained using the Prime Script Reverse Transcriptase System Kit (Takara, Shiga, Japan). Using the PCR primers (F: TGC AAG GCG AAA GAA AGC AG, R: CCC CAA GAT TTG TTC TCC CTG A), we amplified the sequence region by PCR, which spans exons 1 to 5 of the *lhx9* transcript.

### Morphometric analysis

Embryos were positioned in the glycerol on the slides and imaged using a dissecting microscope (Digital sight; Nikon, Tokyo, Japan), using a 3× or 10× objective. Along the anterior-posterior axis, we determined the linear distance from the epiphysis to the tail tip as body length. Then, we outlined the whole eye with the lens to quantify the area of the eye.

### Immunofluorescence staining

Zebrafish embryos were fixed in 4% paraformaldehyde (PFA) for 12 h at 4°C, dehydrated in 30% sucrose overnight at 4°C, embedded in OCT, and cryosectioned at 16 μm thickness. The rest of the procedures for immunofluorescence staining were performed as described in a previous study (Vitorino et al., 2009). The primary antibodies used in this study are listed in Table S1. The number of positive cells in each retina was counted manually using two to four sections per embryo. The width of Zn8^+^ cells was measured as the width of the GCL, using the Adobe Photoshop CC (https://www.adobe.com/cn/products/photoshop.html).

### WISH and section ISH

To block pigmentation, embryos were treated with 0.003% PTU and fixed as described above. *lhx9* specific primers were 5′-GAT GAA CGG ACG GGA GAC-3′ and 5′-GGA GGG TAG GGT TGC TGA-3′ We amplified the target fragment by PCR and synthesized the riboprobe via T7 or T3 RNA Polymerase (Thermo Fisher Scientific, Lafayette, CO, USA). The detailed procedures for WISH and section ISH have been described in previous studies (Balasubramanian et al., 2014; Sheng et al., 2010).

### Visual-motor behavioral test

At 5 dpf, we performed a behavioral test with a Danio Vision system (Noldus Information Technology, Wageningen, Netherlands) to assess the visual function of zebrafish. Larvae from three groups were transferred to a 96-well plate with 200 μl E3 medium per well, one by one. After 30 min in the dark, larvae were subjected to a 30-min light stimulus and their average speed was tracked for 4 min, with the last 2 min in the dark and the first 2 min in the light. To evaluate the light responses, we compared the speed in the last 2 min of the dark with the speed in the first 10 s of the light.

### Quantitative RT-PCR

At 96 hpf, 15 larvae were collected from every group, and cDNA was obtained as described above. The rest of the procedures for qRT-PCR have been described in a previous study (Sheng et al., 2010). The primers used are listed in Table S2.

### Statistics

In this study, statistical analysis was performed using GraphPad Prism software 8.0.1 (https://www.graphpad.com), and one-way analysis of variance (ANOVA) was performed for multiple comparisons. The error bar represents the standard error of the mean (s.e.m.), and ‘*n*’ denotes the number of larvae examined. A *P*-value less than 0.05 was considered statistically significant.

## Supplementary Material

Supplementary information
