# Supplementary material for: LIM Homeobox 9 knockdown by morpholino does not affect zebrafish retinal development
Source: Biol Open. 2021 Mar 8;10(3):bio056382. doi: 10.1242/bio.056382 (PMC7969587; doi:10.1242/bio.056382)
Supplement: Supplementary information [file biolopen-10-056382-s1.pdf]

Supplemental Material

Table S1 Primary antibodies for immunofluorescence staining

Antibody	Dilution	Source	Recognization
Rb anti- GABA	1:500	Sigma	GABAergic ACs
Ms anti- Parvalbumin	1:50	Sigma	One ACs type
Rb anti- Calretinin	1:200	Millipore	One ACs type
Rb anti-TH	1:50	Chemicon	dopaminergic ACs
Ms anti- Zn-8	1:10	DSHB	RGCs
Rb anti- PKCα	1:1000	Sigma	On-BCs
Ms anti- Zpr-1	1:500	Abcam	Red and green cones
Ms anti- Rhodopsin	1:500	Abcam	rods
Rb anti- Activated caspase-3	1:200	R&D Systems	apoptosis cells

Rb anti- Phospho-Histone H3	1:500	Santa Cruz	G2/M-phase proliferation cells

Table S2 Primers for qRT-PCR

Gene name	Primer sequence
prox1 F	5’-ACCATGACAGCACATCCCTC- 3’
prox1 R	5’-ACGTTGGACTTCTCACCGTC- 3’
gs F	5’-CACGTCTGCCAGTTCTCAGT- 3’
gs R	5’-GCCTTCAGCTTGATACGTGC- 3’
nos F	5’-ACCCTGAAGAACGTGTCACC- 3’
nos R	5’-GCCCTGAGCTCTTTGGTCAT- 3’
vsx2 F	5’-TCTTTCTACAGTCAGCCCGC- 3’
vsx2 R	5’-GTGCATCCCTAGAAGCCAGG- 3’
hcrt F	5’- GGACTGCACAGCTAAGAAGC- 3’
hcrt R	5’- AGAGTCGTTTCTGCGTCCTG- 3’
β-actin F	5’- CATGCCATCCTGCGTCT- 3’
β-actin R	5’- AAACGCTCATTGCCGAT- 3’

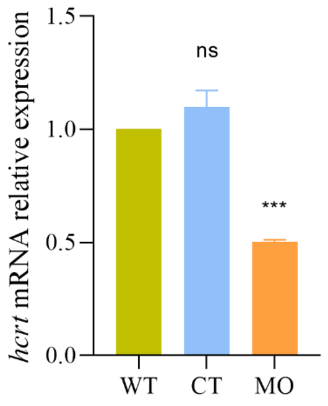

Fig. S1 Effects of *lhx9* knockdown on the expression of *hcrt* in zebrafish at 48 hpf. Results are presented mean ± SEM (n=3 biological replicates). ns, $P > 0.05$; ***, $P < 0.001$; assessed by one-way ANOVA followed by Tukey's multiple comparisons.

Biology Open • Supplementary information

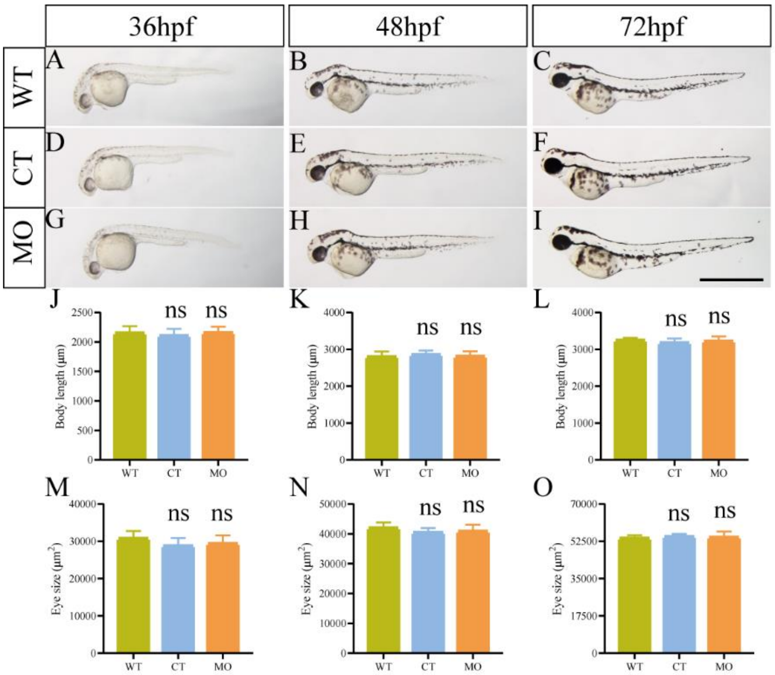

Fig. S2 Effects of *lhx9* knockdown on the zebrafish morphology. A-I, Lateral view of WT (A-C), CT (D-E), MO (G-I) embryos at 36 hpf, 48 hpf, and 72 hpf. Scale bar = 1000 μm. J-O, Graphic analysis of body length (J-L) and eye size (M-O) of embryos. Results are presented as the mean±SEM (WT, n = 15; CT, n = 16; MO, n = 16). ns, $P > 0.05$; assessed by one-way ANOVA followed by Tukey's multiple comparisons.

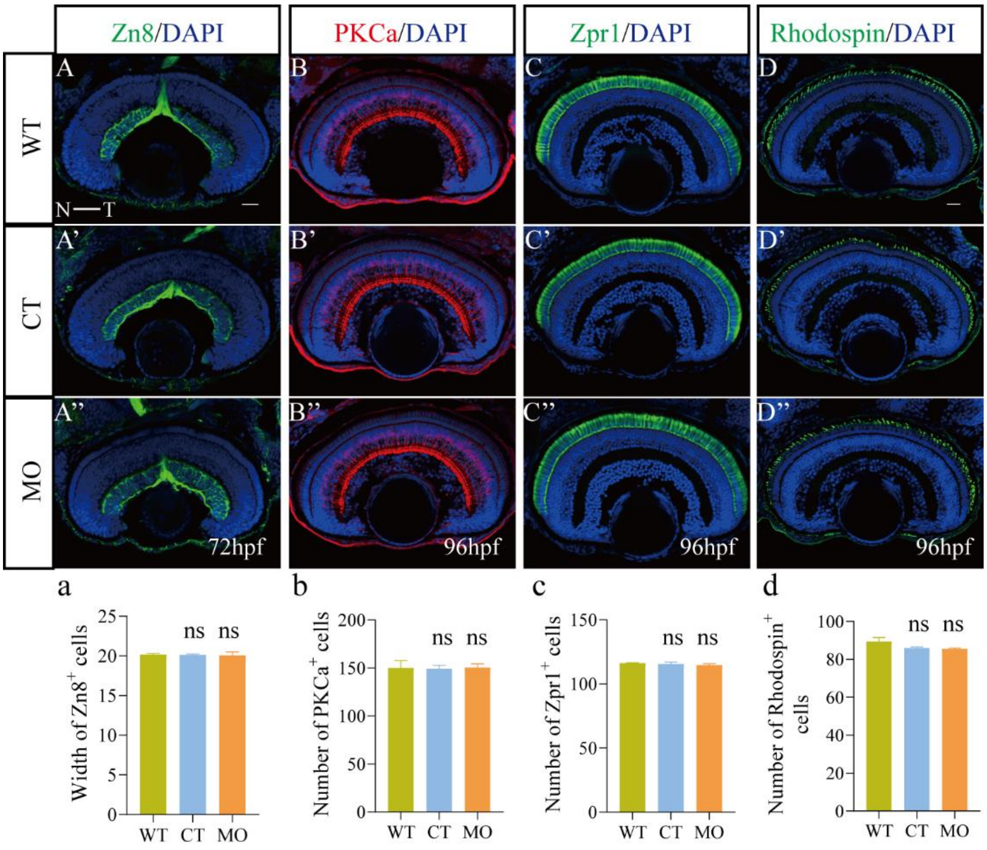

Fig.S3 Immunofluorescence staining analysis of the retinal neuronal differentiation. All figures are horizontal sections along the temporal-nasal axis (T-N). A-D'', Immunofluorescence staining with other retinal neural markers, Zn8, PKCα, Zpr1, and Rhodospin, in WT (A-D), CT (A'-D'), and MO (A''-D'') retinas. a-d, statistical analysis of Zn8^{+}, PKCα^{+}, Zpr1^{+}, and Rhodospin^{+} cells in WT, CT, and MO retinas. A-A'', at 72 hpf; others, at 96 hpf. Blue, DAPI staining of the nuclei. Scale bar =20 µm. a-d, Statistical analysis of the positive cells in WT, CT, and MO retinas at 36 hpf and 48 hpf. Results are presented as the mean SEM (WT, n = 16; CT, n = 15; MO, n = 16). ns, $P > 0.05$; assessed by one-way ANOVA followed by Tukey's multiple comparisons.

Biology Open • Supplementary information

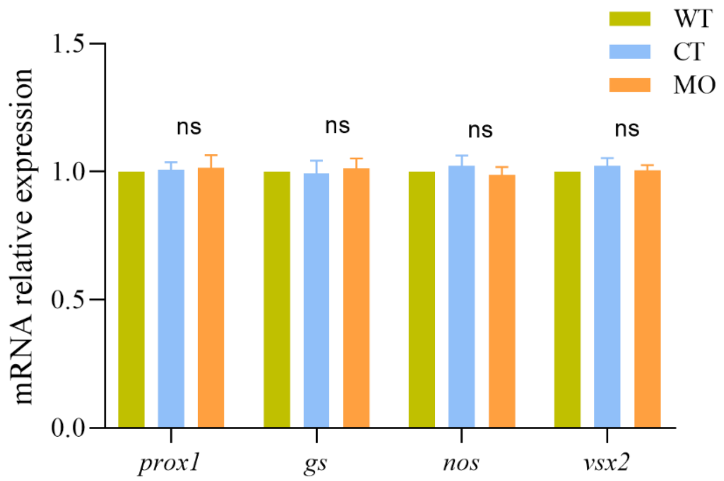

Fig.S4 qRT-PCR analysis of the expression of the retinal genes at 96hpf. Results are presented mean ± SEM (n = 3 biological replicates). ns, $P > 0.05$; assessed by one-way ANOVA followed by Tukey's multiple comparisons.

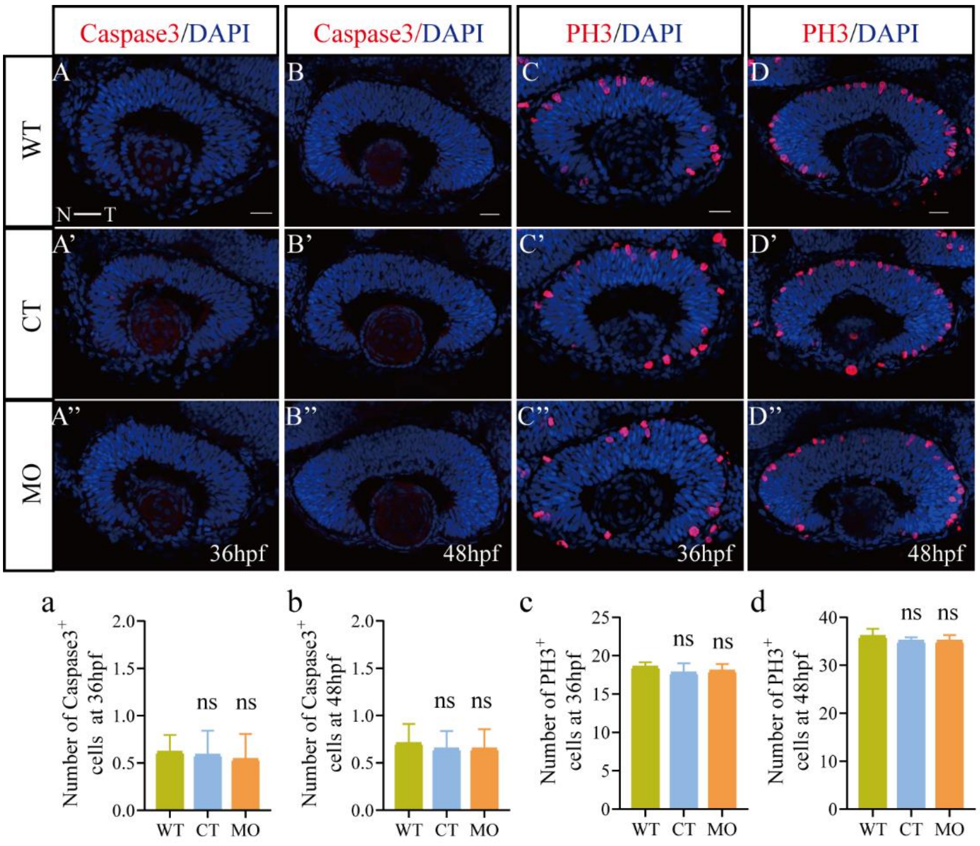

Fig.S5 Effects of *lhx9* knockdown via MO on the apoptosis and proliferation in the retina. All figures are horizontal sections along the temporal-nasal axis (T-N). A-B'', Immunofluorescence staining with Caspase3 at 36 hpf (A-A'') and 48 hpf (B-B''). C-

Biology Open • Supplementary information

D'', Immunofluorescence staining with PH3 at 36 hpf (C-C'') and 48 hpf (D-D''). Scale bar =20μm. a-d, Statistical analysis of Caspase3^{+} and PH3^{+} cells in WT, CT, and MO retinas at 36hpf and 48hpf. Results are represented as the mean±SEM (WT, n = 12; CT, n = 17; MO, n = 15). ns, $P > 0.05$; assessed by one-way ANOVA followed by Tukey's multiple comparisons.

Biology Open • Supplementary information
